# From Inflammation to Thrombosis: The Prothrombotic State and Cardiovascular Risk in Inflammatory Bowel Disease

**DOI:** 10.3390/medicina62020270

**Published:** 2026-01-27

**Authors:** Vlad Dumitru Brata, Dana Alina Crisan, Angela Cozma, Cezara-Andreea Gerdanovics, Stefan Lucian Popa, Mircea Vasile Milaciu, Olga Hilda Orășan

**Affiliations:** 1 Department of Gastroenterology, Regional Institute of Gastroenterology and Hepatology “Prof. Dr. Octavian Fodor”, 400394 Cluj-Napoca, Romania; 2 Department of Internal Medicine, “Iuliu Hațieganu” University of Medicine and Pharmacy, 400012 Cluj-Napoca, Romania; 3 Clinical Municipal Hospital, 400139 Cluj-Napoca, Romania; 4 Department of Internal Medicine, 4th Medical Discipline, “Iuliu Hațieganu” University of Medicine and Pharmacy, 400015 Cluj-Napoca, Romania; angelacozma@yahoo.com (A.C.);; 5 Second Medical Department, “Iuliu Hatieganu” University of Medicine and Pharmacy, 400000 Cluj-Napoca, Romania

**Keywords:** inflammatory bowel disease, venous thromboembolism, cardiovascular risk, thrombo-inflammation, therapeutic agents, risk stratification

## Abstract

Inflammatory bowel disease (IBD) is associated with an increased risk of venous thromboembolic events (VTEs) and a moderate risk of arterial cardiovascular events. This varies with inflammatory activity and acute-care exposure, with pathophysiological data supporting a thromboinflammatory phenotype in which intestinal inflammation influences systemic vascular homeostasis through innate immune activation, coagulation–platelet crosstalk, endothelial dysfunction, impaired fibrinolysis, and immunothrombosis. Clinically, prevention and management should be integrated into routine care and anchored in sustained, steroid-sparing disease control, combined with guideline-based in-hospital thromboprophylaxis and standard cardiovascular prevention. Decisions regarding anticoagulant therapy after VTEs should follow established principles while recognizing that recurrence prevention depends not only on anticoagulant choice but also on minimizing repeated inflammatory and treatment-related risk exposures. Cardiovascular risk assessment and optimization of modifiable factors should be considered before therapy escalation or treatment switching. Future advances will likely come from more personalized risk assessment across dynamic high-risk windows and from adjunctive, mechanism-informed strategies targeting key nodes of the gut–vascular interface and immunothrombosis.

## 1. Introduction

Inflammatory bowel disease (IBD) represents a group of chronic, immune-mediated diseases of the gastrointestinal system, encompassing mainly Crohn’s disease (CD) and ulcerative colitis (UC). These conditions are characterized by distinct pathophysiological and clinical features, sharing complex etiological mechanisms involving genetic predisposition, immune dysregulation, environmental factors, and gut microbiota alterations [1,2].

Beyond intestinal manifestations, extraintestinal involvement is common and substantially contributes to disease burden. Cardiovascular involvement, while often less emphasized than musculoskeletal or dermatologic manifestations, is increasingly supported by population-level evidence and pathophysiological data [2,3]. Among systemic complications, venous thromboembolism (VTE) and arterial cardiovascular events represent major contributors to morbidity and mortality. Risk is strongly modulated by disease activity and clinical context (e.g., flare-ups, hospitalization, perioperative periods) [4,5].

The association between IBD and thrombosis is clinically relevant because vascular events occur in a population in which traditional cardiovascular risk profiles may not fully account for observed event rates [5,6]. In large cohorts, cardiovascular risk has been shown to vary over time and to rise during periods of active disease and around acute-care exposures, including hospitalization and surgery [7,8].

Pathophysiological studies support a link between intestinal inflammation and systemic vascular vulnerability. Pro-inflammatory pathways in IBD involve cytokines such as TNF-α and IL-6, and Th17-related signaling, which can contribute to endothelial activation, oxidative stress, and impaired vascular homeostasis [4,7,9]. Systemic inflammation also reshapes hemostatic balance, and abnormalities in platelet activation and in the coagulation–fibrinolysis balance have been described in IBD, consistent with a prothrombotic phenotype that may intensify during flares [9,10].

These observations have therapeutic implications. International consensus guidance recommends pharmacologic thromboprophylaxis during hospitalization for patients with IBD in the absence of contraindications, and emphasizes disease activity as a modifiable risk factor, supporting treatment strategies aimed at sustained, steroid-sparing remission [5]. Standard cardiovascular prevention—blood pressure and lipid management, smoking cessation, physical activity, and weight optimization—should be incorporated into routine care. Treatment selection and monitoring should also consider drug-specific safety profiles relevant to thrombosis and major adverse cardiovascular events [5].

The aim of this narrative review is to provide an overview of the epidemiology and magnitude of thrombotic and cardiovascular risk in patients with IBD, outlining the underlying pathophysiological mechanisms linking intestinal inflammation and vascular dysfunction, and to discuss the impact of IBD management on the overall thrombotic and cardiovascular risk of these patients. Accordingly, this review takes an integrative approach, linking population-level risk estimates, pathophysiological mechanisms, and prevention and management considerations.

## 2. Literature Search

We performed a structured literature search across PubMed/MEDLINE and Embase from their inception to 15 December 2025. The following search strings were used to navigate the literature on (i) thrombotic and cardiovascular risk and mechanisms, and (ii) management and future directions:

(i). (“inflammatory bowel disease” OR “IBD” OR “Crohn’s disease” OR “ulcerative colitis”) AND (“thrombosis” OR “venous thromboembolism” OR “VTE” OR “deep vein thrombosis” OR “DVT” OR “arterial thrombosis” OR “cardiovascular risk” OR “ischemic heart disease” OR “myocardial infarction” OR “stroke” OR “atrial fibrillation” OR “MACE” OR “PAD” OR “peripheral artery disease”) AND (“inflammation” OR “cytokines” OR “tissue factor” OR “platelets” OR “endothelial dysfunction” OR “fibrinolysis” OR “neutrophil extracellular traps” OR “NETs” OR “immunothrombosis” OR “TLR4” OR “lipopolysaccharide”).

(ii). (“inflammatory bowel disease” OR “IBD” OR “Crohn’s disease” OR “ulcerative colitis”) AND (“thromboprophylaxis” OR “prophylaxis” OR “anticoagulation” OR “direct oral anticoagulant” OR “direct oral anticoagulants” OR “DOAC” OR “low molecular weight heparin” OR “LMWH” OR “guidelines” OR “consensus” OR “perioperative” OR “postoperative” OR “post-discharge” OR “prevention” OR “management” OR “corticosteroids” OR “steroid sparing” OR “biologics” OR “anti-TNF” OR “vedolizumab” OR “ustekinumab” OR “JAK inhibitors” OR “tofacitinib” OR “risk stratification” OR “risk prediction” OR “biomarkers”) AND (“thrombosis” OR “venous thromboembolism” OR “VTE” OR “cardiovascular risk” OR “MACE”).

Duplicate records were removed prior to screening. Reference lists of included articles and relevant reviews were hand-searched to identify additional studies of potential relevance.

## 3. Epidemiology and Magnitude of Risk

IBD is associated with a higher risk of VTE compared with the general population, varying with disease activity and clinical context. Data suggest that the risk of VTE is approximately twofold higher compared to the general population, observed in both CD and UC [6]. In a large UK cohort, the overall VTE hazard ratio (HR) was 3.4 (95% CI 2.7–4.3), with an absolute risk of 2.6 events per 1000 person-years, and a significantly increased risk during flares (HR = 8.4, 95% CI 5.5–12.8, with an absolute risk of 9 per 1000 person-years) [8]. Recent nationwide data from a Swedish cohort confirm these findings; the incidence was 5.03 per 1000 person-years, with an HR of 2.12 (95% CI 2.02–2.23), compared with 2.35 per 1000 person-years in matched controls [11]. The incidence of VTE increases during active disease and decreases during remission, supporting inflammatory activity as a key determinant of thrombotic risk [8]. Notably, during flares, the relative risk was higher in ambulatory periods (RR = 15.8; 95% CI 9.8–25.5) than during hospitalization (RR = 3.2; 95% CI 1.7–6.3), but the absolute risk reached 37.5 per 1000 person-years during hospitalized flares in this cohort [8].

Nevertheless, relative and absolute risk should be interpreted together, as absolute rates are highest during hospitalized flares [8,12]. This difference is clinically significant for risk stratification and prophylaxis decisions. Relative risk quantifies the proportional excess risk attributable to IBD, which may appear highest in younger patients, while absolute risk reflects the event burden in specific clinical states and, thus, the likely impact of preventive strategies. Although additional cohort data have accumulated, VTE risk estimates remain heterogeneous across studies, and this must be explicitly considered when interpreting the magnitude of association. The variation likely reflects differences in population case-mix (ambulatory vs. hospitalized patients, perioperative contexts), outcome definitions, and healthcare system factors influencing hospitalization thresholds and prophylaxis guidelines, as well as analytic handling of time-varying exposures, such as hospitalization, surgery, and medication use. Recent population-based studies further suggest that, despite evolving IBD management and guideline implementation, VTE incidence has not uniformly declined, reinforcing the importance of context when comparing cohorts, rather than assuming a single stable IBD-related effect [13].

Age-specific differences are also relevant, as the absolute VTE risk increases with age, while the relative risk is higher in younger patients, with an HR of 6.0–6.4 for VTE compared to age-matched controls [14]. The difference between absolute and relative risk is mainly due to the fact that baseline VTE rates in the general population increase with age. Thus, the same disease-associated absolute increase can translate into a larger proportional effect in younger patients, whereas older patients accumulate the highest absolute burden. From a clinical perspective, younger patients exhibit the greatest proportional excess risk attributable to IBD (particularly when traditional thrombotic risk factors are less prevalent), while older patients require increased attention because absolute event rates are highest during high-risk phases, such as hospitalization and perioperative care.

This has been confirmed by Asian cohorts as well, with VTEs of 15.26 per 1000 person-years during hospitalized flare, with an incidence of 14.53 per 1000 person-years in young patients and 34.58 in older patients [12]. Sex-specific differences appear limited overall, with similar risk across large cohort studies and meta-analyses [14,15]. However, a lack of reported sex differences in registry-based analyses does not necessarily exclude clinically relevant effect modification. Sex-linked exposures and situations (e.g., hormonal factors and pregnancy/post-partum periods) are not consistently accounted for and may attenuate true differences. In contrast, pooled evidence for arterial events suggests a more consistent association in women than in men, indicating that sex-related patterns may differ between VTE and arterial cardiovascular outcomes in IBD [10].

VTE in IBD tends to occur at a younger age than in the general population, with recurrence being a significant concern [16]. In a cohort focused on recurrence after a first VTE, the 5-year probability of recurrence after stopping anticoagulation was 33.4% (95% CI 21.8–45.0) in IBD versus 21.7% (95% CI 18.8–24.6) in non-IBD patients [16]. Risk is not distributed evenly across clinical settings. Hospitalization and the perioperative period represent high-risk contexts in which inflammatory activity frequently coexists with acquired triggers such as reduced mobility, dehydration, infection, and procedural/surgical stress [8,17]. A recent meta-analysis concluded that the strongest risk factors associated with VTE in IBD patients were prior VTEs and surgery-related factors, including postoperative/surgical complications [17]. Accordingly, international consensus guidance recommends pharmacologic thromboprophylaxis during hospitalization in the absence of contraindications and emphasizes disease activity as a modifiable risk factor in overall thrombotic risk reduction [5].

Apart from VTE, IBD is associated with a modest increase in arterial cardiovascular events, including ischemic heart disease and cerebrovascular events. Across pooled data, the magnitude of association has been estimated at an OR of 1.19 (95% CI 1.08–1.31) for ischemic heart disease and an OR of 1.18 (95% CI 1.09–1.27) for cerebrovascular accidents, with a more consistent signal in women than in men [10]. Large cohort studies further suggest an activity-dependent risk for myocardial infarction (MI) and stroke, rising during flares, with MI RR = 1.49; stroke RR = 1.53, and persistent activity, with MI RR = 2.05; stroke RR = 1.55, while the risk was comparable to the general population during periods of remission [18].

Furthermore, an increased risk of atrial fibrillation (AF) has also been observed during the active stages of the disease, while this association was less evident during remission. In Danish registry data, AF incidence was 4.16 vs. 2.70 per 1000 person-years in IBD versus controls (overall IRR = 1.26, 95% CI 1.16–1.36), driven by flares (IRR = 2.63, 95% CI 2.26–3.06) and persistent activity (IRR = 2.06, 95% CI 1.67–2.55), with no excess during remission (IRR = 0.97, 95% CI 0.88–1.08) [19].

Confounding by disease severity and corticosteroid exposure represents a fundamental challenge in interpreting observational associations between IBD and VTE, because steroid initiation is closely linked to greater inflammatory burden and the highest baseline thrombotic risk. This challenge can be synthesized across several interrelated dimensions. First, IBD-focused evidence syntheses support an independent corticosteroid signal, with systemic corticosteroid exposure consistently associated with increased VTE risk compared with non-steroid regimens and biologic-based strategies [20]. Second, confounding by indication complicates causal attribution because corticosteroids are preferentially initiated during severe flares—the clinical state most strongly linked to thrombosis—and because disease activity and cumulative steroid dose/duration may be incompletely captured in registry datasets, leaving residual confounding even after adjustment. Third, comparative therapeutic data help differentiate medication effects from disease severity: anti-TNF therapy is associated with a lower VTE risk compared to corticosteroids, supporting a direct pharmacological effect beyond confounding by indication alone [21]. Notably, combination therapy with corticosteroids and biologics attenuates this apparent benefit, with VTE risk approaching that observed with corticosteroid exposure [22]. Finally, temporality-sensitive evidence supports a direct drug contribution, with the highest VTE risk occurring shortly after initiation of systemic glucocorticoids and remaining elevated during exposure periods [23]. Taken together, these observations support the interpretation that corticosteroids contribute to an independent prothrombotic risk that cannot be fully explained by confounding by disease severity alone.

Overall, these observations support an activity-dependent vascular risk profile in IBD, in which thrombotic and cardiovascular events cluster during periods of heightened inflammatory activity and are not fully accounted for by traditional cardiovascular risk factors, particularly in younger patients and women [10,18,19]. This pattern suggests that immune-mediated and activity-associated mechanisms—rather than classic atherosclerotic pathways alone—play a central role in vascular risk in IBD.

## 4. The Prothrombotic State in IBD: Phenotype and Mechanisms

**Figure 1:**
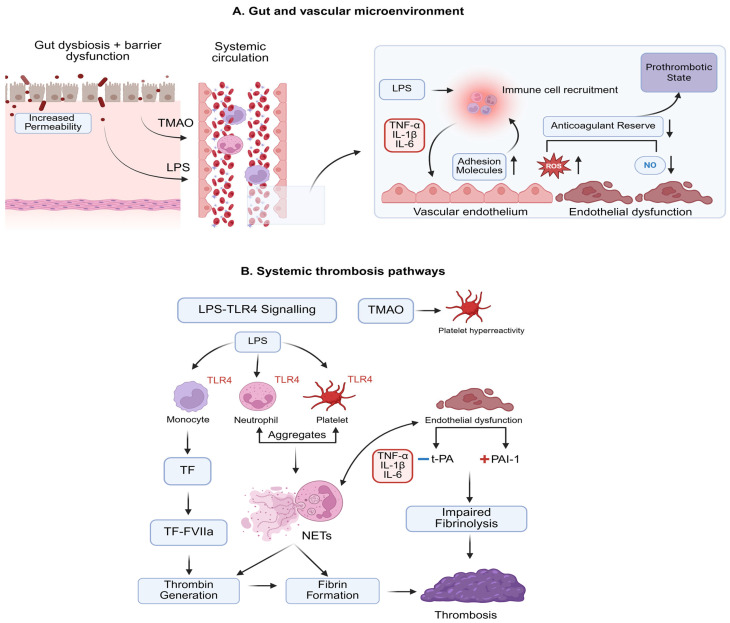
Gut–vascular axis in IBD: pathways contributing to a prothrombotic phenotype. (**A**) IBD-associated gut dysbiosis promotes barrier dysfunction, resulting in increased gut permeability and translocation of microbial products into the systemic circulation, including bacterial LPS and gut-derived metabolites (e.g., TMAO). Circulating LPS drives the recruitment of immune cells, together with the production of pro-inflammatory cytokines, such as TNF-α, IL-1β, and IL-6. These mediators promote endothelial activation with upregulation of adhesion molecules, sustaining leukocyte adhesion and promoting further inflammation. In parallel, endothelial dysfunction and inflammatory signaling induce the production of ROS and a reduction in NO bioavailability, contributing to endothelial dysfunction and a reduced endogenous anticoagulant reserve, establishing a prothrombotic vascular microenvironment. (**B**) LPS-TLR4 signaling links microbial translocation to thrombo-inflammation by activating monocytes, neutrophils, and platelets expressing TLR4. In monocytes, this activation promotes TF expression, initiating the extrinsic coagulation pathway through activating F VII and subsequent thrombin generation and fibrin formation. In parallel, LPS- and cytokine-driven platelet activation promotes platelet–neutrophil aggregate formation and amplifies leukocyte–platelet crosstalk, creating conditions that favor NET release. NETs contribute to immunothrombosis by providing a structural scaffold that traps platelets and erythrocytes, concentrates procoagulant proteins, and stabilizes fibrin-rich thrombi, thereby reinforcing thrombus propagation. Endothelial dysfunction further sustains thrombosis by shifting the hemostatic balance toward clot persistence through impaired fibrinolysis, reflected by reduced t-PA and increased PAI-1. Together, TF-dependent coagulation, platelet–leukocyte interactions, NET-mediated immunothrombosis, and impaired fibrinolysis converge on thrombosis. Abbreviations: IBD—inflammatory bowel disease; LPS—lipopolysaccharides; TMAO—trimethylamine-N-oxide; TNF-α—tumor necrosis factor alpha; IL-1β—interleukin 1β; IL-6—interleukin 6; ROS—reactive oxygen species; NO—nitric oxide; TLR4—Toll-like receptor 4; TF—tissue factor; F VIIa—activated coagulation factor number VII; NETs: neutrophil extracellular traps; t-PA—tissue plasminogen activator; PAI-1—plasminogen activator inhibitor-1.

The prothrombotic state in IBD is multifactorial and closely linked to disease activity, reflecting several distinct but interconnected pathophysiological mechanisms that reshape coagulation, platelet activity, and vascular homeostasis. A logical cascade can be traced from gut dysbiosis and barrier dysfunction to systemic exposure to microbial and inflammatory mediators, followed by coordinated activation of tissue factor-dependent coagulation, platelet–leukocyte interactions, endothelial dysfunction, and impaired fibrinolysis—ultimately lowering the threshold for venous and, to a lesser extent, arterial thrombosis [24,25]. Figure 1 summarizes a proposed gut–vascular cascade linking dysbiosis and barrier dysfunction to endothelial activation, immunothrombosis, and impaired fibrinolysis in IBD.

Gut dysbiosis is a key pathophysiological feature of IBD, encompassing altered microbiota composition and increased intestinal permeability, which facilitates translocation into the systemic circulation of microbial products, such as bacterial endotoxins and lipopolysaccharides (LPSs). Circulating LPS activates the Toll-like receptor 4 (TLR4) signaling pathway in leukocytes, endothelial cells, and platelets, promoting inflammation and shifting vascular and cellular responses toward a procoagulant status [26,27,28]. Additionally, preclinical evidence suggests that gut dysbiosis may be linked to prothrombotic gut-derived metabolites, such as trimethylamine-N-oxide (TMAO). In experimental models, TMAO enhances platelet hyperreactivity and thrombus formation [29]. In IBD, recent human studies have begun to explore TMAO-related immuno-metabolic pathways and vascular phenotypes, supporting the plausibility of this potential microbiota-dependent link, distinct from LPS-TLR4 signaling. However, direct evidence connecting TMAO to clinical thrombotic events in IBD is currently limited, and this pathway requires further validation in human cohorts [28,29,30].

One central pathophysiological link between inflammation and thrombosis is tissue factor (TF). LPS binding to TLR4 induces TF expression in monocytes, initiating the extrinsic coagulation pathway via factor VII/VIIa and promoting thrombin generation and fibrin formation [31,32]. Moreover, recent early-stage evidence indicates that colitogenic CD4+ T cells can express TF and exhibit TF-dependent thrombogenicity. This has been demonstrated in experimental colitis models and supported by the detection of TF+ CD4+ T cells in human IBD samples, identifying an emerging inflammation-linked source of procoagulant activity. Nevertheless, the clinical significance of T cell-associated TF and its contribution to thrombotic risk remain to be fully established [32,33].

Pro-inflammatory mediators promote endothelial activation, including upregulation of adhesion molecules and further leukocyte recruitment, increased production of reactive oxygen species (ROS), and decreased nitric oxide (NO) bioavailability, collectively shifting the endothelium towards a procoagulant status. Microvascular endothelial dysfunction has been demonstrated in IBD, consistent with impaired vascular homeostasis during chronic inflammation [9]. Endothelial perturbation also affects endogenous anticoagulant mechanisms at the endothelial interface, reinforcing the concept that active inflammation lowers anticoagulant reserve and promotes thrombin generation through multiple, converging pathways [9].

Furthermore, TLR4 signaling on platelets enhances their activation, aggregation, and formation of platelet–leukocyte aggregates, stimulated by TNF-α, IL-1β, and IL-6 [34,35]. Experimental data also support the role of platelet TLR4 signaling in regulating platelet activation and function in inflammatory settings, providing a plausible mechanistic route by which endotoxin exposure can facilitate platelet-driven thrombosis, although direct evidence linking platelet TLR4 signaling to thrombotic outcomes in human IBD remains limited [36]. In addition, pro-inflammatory mediators and endothelial injury reduce tissue plasminogen activator (t-PA) and increase the activity of plasminogen activator inhibitor-1 (PAI-1), leading to decreased fibrinolysis [37].

Neutrophil extracellular traps (NETs) are an increasingly recognized contributor to the prothrombotic state in IBD, supported by evidence from experimental models and human observational studies [38]. NETs are web-like extracellular structures composed of decondensed DNA decorated with histones and neutrophil granule proteins (including neutrophil elastase and myeloperoxidase), released during neutrophil activation [39]. In IBD, chronic intestinal inflammation and dysbiosis promote neutrophil recruitment and activation, creating conditions that favor excessive NET formation [38]. NET release can be induced by microbial products such as LPS—either directly through neutrophil sensing of LPS or indirectly through platelet–neutrophil interactions mediated by TLR4-dependent platelet activation—and is also promoted by inflammatory cytokine signaling, including TNF-α and IL-1β [39,40].

Once formed, NETs support thrombosis through several complementary mechanisms. They provide a physical scaffold that traps platelets and erythrocytes and concentrates procoagulant proteins, thereby stabilizing developing thrombi and supporting fibrin deposition [38]. NET-associated histones can enhance platelet adhesion/activation and can injure or activate the endothelium, further shifting the vessel wall toward a proadhesive, procoagulant phenotype [39]. In addition, preclinical and ex vivo data indicate that NETs can amplify coagulation by providing procoagulant surfaces and facilitating clotting reactions, including contact pathway activation, although the magnitude and clinical relevance of these mechanisms for thrombotic events in human IBD remain to be clarified [41]. These effects position NETs as an immune-driven propagation layer that complements tissue factor-dependent thrombin generation, platelet activation, and impaired fibrinolysis, and provides a plausible mechanistic link to why thrombotic risk in IBD increases during periods of heightened inflammatory activity [38,39,40,41].

These mechanisms converge to create a coherent, prothrombotic phenotype in IBD. Gut-derived innate immune activation (LPS–TLR4), TF-dependent thrombin generation (including TF+ immune cells), endothelial dysfunction, platelet–leukocyte interactions, impaired fibrinolysis, and NET-mediated immunothrombosis act in parallel and reinforce one another, particularly during periods of active disease. This framework explains why thrombotic and cardiovascular events cluster around flares and acute care exposures, often independent of traditional cardiovascular risk profiles.

The prothrombotic mechanisms in IBD can be stratified by the level of validation in human studies. Pathways supported most consistently by human clinical and laboratory observations include gut-derived innate immune activation (including LPS-TLR4 signaling), monocyte-derived TF activity with thrombin generation, platelet activation with platelet–leukocyte aggregate formation, endothelial dysfunction with loss of anticoagulant reserve, and impaired fibrinolysis through increased PAI-1 activity [26,27,28,31,32,34,35,36,37]. NET formation and NET-associated immunothrombotic signatures are increasingly supported by experimental models and human observational studies in IBD, with markers tracking inflammatory activity, although the extent to which specific NET-driven coagulation mechanisms translate into clinical thrombotic events remains to be established [38]. Upstream or cell-specific mediators such as TMAO-related platelet effects and TF expression on colitogenic CD4+ T cells should be interpreted as emerging pathways- biologically plausible and supported by experimental data, but requiring further validation to determine their contribution to clinical thrombotic events and their relevance to established TF-and inflammation-driven mechanisms [28,29,30,33].

## 5. Cardiovascular Manifestations and Risk Beyond VTE

Beyond VTE, IBD has been associated with a modest but clinically relevant excess risk of cardiovascular outcomes, including AF, ischemic heart disease, MI, stroke, and potentially peripheral artery disease (PAD). Although conventional risk factors (e.g., smoking, metabolic comorbidity) contribute to absolute event rates, they do not consistently explain the observed associations, which appear to reflect disease-specific risk modifiers, including inflammatory burden, treatment exposure, and acute-care stressors [4,9,18]. Periods of active disease and acute care exposures (hospitalization, surgery) often represent higher-risk contexts, supporting an activity-associated vascular vulnerability. Table 1 summarizes the major studies assessing the cardiovascular risk beyond VTE in IBD patients, highlighting the magnitude of association across key outcomes.

A series of studies report an association between IBD and AF, with AF incidence being increased during flares and persistent disease activity, but not during remission [19,45]. Chronic inflammation may promote atrial remodeling and autonomic imbalance, with oxidative stress contributing to fibrosis and electrophysiologic instability [46]. Conduction abnormalities have also been described, including prolonged atrial conduction times [47]. These observations support an activity-dependent arrhythmic risk signal, rather than a uniform excess risk across all disease stages.

Coronary involvement in IBD is heterogeneous and likely reflects the convergence of inflammation-driven vascular injury and conventional atherosclerotic pathways. Systemic inflammation can promote endothelial dysfunction, platelet activation, and a procoagulant shift. These changes may interact with traditional risk factors and precipitate atherothrombotic events, particularly during flares and acute-care exposures (hospitalization, surgery) [9,18]. Importantly, both clinical and histologic activity have been linked to major adverse cardiovascular outcomes, supporting inflammatory burden as a disease-specific modifier of coronary risk in IBD [48,49].

Consistent with this concept, cohort data suggest a modest excess risk of ischemic heart disease and MI, with several analyses reporting stronger relative associations in women and younger patients [48]. In Danish nationwide data, MI and cardiovascular death risks were highest during flares and persistent activity and were not increased during remission, reinforcing that coronary risk is not constant over time but clusters during periods of active disease [18]. A large U.S. electronic health record analysis similarly reported higher MI rates in IBD, with particularly high relative estimates in younger adults and women; however, the higher prevalence of traditional risk factors in the IBD group underscores that absolute risk is shaped by both inflammatory activity and background cardiometabolic risk [49]. These patterns are most consistent with inflammation lowering the threshold for acute coronary syndromes in predisposed individuals, rather than implying an unavoidable, lifelong coronary risk excess in all patients with IBD [9,18].

Recent evidence also reports an association between IBD and ischemic stroke, with disease activity and other comorbidities mitigating the risk [18,19,50,51]. Moreover, there are some studies in which the stroke risk has been higher in patients with CD than UC [52]. AF occurring during active IBD may also contribute to stroke risk in selected patients, highlighting a clinically relevant overlap between arrhythmic and cerebrovascular phenotypes in inflammatory states [19]. In practice, these observations reinforce the importance of recognizing transient risk clustering and ensuring timely evaluation of new neurologic symptoms during flares and high-inflammatory periods.

While the relationship between IBD and AF, MI, ischemic heart disease, and stroke has been studied and confirmed by large population-based studies, evidence regarding the connection between IBD and PAD is less consistent. Some population-based studies reported an increased PAD risk in IBD, with stronger signals in patients with frequent IBD-related hospitalizations, suggesting an effect of disease severity and cumulative inflammatory burden [53]. However, pooled estimates have been heterogeneous and have not consistently demonstrated a significant overall association. Accordingly, PAD should be regarded as a less settled cardiovascular phenotype in IBD compared with AF, MI, and ischemic stroke [10]. Future prospective studies are needed to clarify the association and identify higher-risk subgroups.

## 6. Impact of Treatment on Thrombotic and Cardiovascular Risk

The primary aim of IBD therapy is to control intestinal inflammation and maintain sustained, steroid-free remission. Because thrombotic and cardiovascular risk in IBD is strongly activity-dependent, the net vascular impact of treatment is shaped by two interacting components: the ability to reduce inflammatory burden and prevent high-risk exposures (e.g., hospitalization, surgery, prolonged immobilization), and drug-specific safety signals, particularly for VTE and major adverse cardiovascular events [22,54]. Importantly, comparisons across drug classes are vulnerable to confounding by indication, since systemic corticosteroids and rescue strategies are preferentially used in patients with severe disease, which itself increases thrombotic risk [22,54]. In this context, systemic corticosteroid exposure shows the most consistent association with increased VTE risk, whereas lower VTE rates have been reported in cohorts treated with anti-TNF agents [54]. For newer agents such as JAK inhibitors, thrombotic risk is generally interpreted as dose- and patient-profile dependent [55]. The main therapeutic agents used in IBD and their effects on the cardiovascular system are detailed in Table 2.

When it comes to aminosalicylates (5-ASA), in patients with IBD treated with 5-ASA, ex vivo and in vitro work revealed reduced platelet activation, including reduced spontaneous and thrombin-induced platelet activation, independent of disease activity [56].

Importantly, several associations between the impact of treatment on cardiovascular risk are based on observational data and may be confounded by disease activity and treatment selection. This is particularly relevant for 5-ASA agents, where reports of lower IHD may reflect residual confounding rather than a direct drug effect [57].

Systemic corticosteroids represent the most consistent treatment-associated risk factor for VTE, being associated with almost a threefold increase in VTEs in these patients, particularly among new users [58]. In a large cohort study comparing treatment categories, VTE occurrence was substantially higher in patients receiving systemic corticosteroids (alone or combined with biologics) than in those receiving biologic therapy alone, with an approximate five-fold difference reported for corticosteroids versus biologic-only treatment [22]. Beyond VTE, prolonged or repeated systemic corticosteroid exposure can worsen cardiovascular risk indirectly through hypertension, dyslipidemia, insulin resistance, and persistent inflammatory activity when steroids are used beyond induction. Clinically, these data support early escalation to steroid-sparing maintenance strategies and minimization of cumulative systemic steroid burden [59].

Among immunomodulators, in vitro data suggest that azathioprine can reduce platelet activation [60]. Large clinical studies investigating azathioprine, mercaptopurine, and methotrexate in IBD patients have not demonstrated an increased risk of VTE [61,62,63]. In a nationwide Spanish cohort of 3391 IBD patients, no VTEs were reported among thiopurine users after a median follow-up of 44 months [61]. Likewise, a prospective study assessing thiopurine tolerability documented no VTEs after a median of 32 months [62]. Consistently, in a study evaluating methotrexate use following thiopurine therapy, none of the 174 CD patients developed VTE during a median follow-up of 10 months [63]. Overall, available data suggest a largely thrombotically neutral profile for conventional immunomodulators, while acknowledging that event rates are low and surveillance intervals vary across cohorts.

Due to its involvement in the pathogenesis of VTEs, TNF-α inhibition can significantly improve the hypercoagulable state in IBD patients, with studies reporting a lower VTE occurrence compared with systemic corticosteroids [54,64]. However, when anti-TNF therapy is compared with other non-steroid regimens, the observed differences are less consistent, likely reflecting heterogeneous baseline risk, disease severity, and differential corticosteroid exposure, reinforcing the dominant role of inflammatory control as a confounding factor and mediator.

The thrombotic and cardiovascular safety profile of vedolizumab—an α4β7 integrin inhibitor with gut-selective activity—has been evaluated across clinical trials and real-world cohorts in adult IBD populations. In pooled safety data from pivotal clinical trials, no excess VTE signal was identified [65]. Similarly, real-world data have generally reported comparable VTE risk to anti-TNF therapy [66,67,68,69]. In older adults, a propensity score-matched comparative study reported a similar risk of major adverse cardiovascular and venous thromboembolic events with vedolizumab versus TNF antagonists (aHR = 0.9, 95% CI 0.41–2.01) [70]. More recently, Japanese real-world data in UC suggested a potentially favorable profile, with vedolizumab associated with a lower VTE risk compared with TNF inhibitors (HR = 0.50; 95% CI 0.30–0.81) [68]. However, in pediatric IBD, a target emulation study reported higher VTE risk in vedolizumab versus anti-TNF therapy (HR = 8.63) and versus ustekinumab (HR = 4.64) in pediatric-onset CD, but not in UC, though baseline disease severity and treatment selection patterns may differ in this population, warranting caution and further studies in this subgroup [71]. Overall, vedolizumab demonstrates a generally favorable thrombotic safety profile in adults, while pediatric CD signals require replication and careful contextualization.

Longer-term pooled safety analyses in IBD clinical programs support a generally favorable cardiovascular and thrombotic safety profile for ustekinumab, targeting interleukins 12 and 23. Comparative observational analyses have not consistently shown an increased VTE risk versus anti-TNF therapy, though head-to-head evidence remains limited and residual confounding is expected [72]. Moreover, data from pediatric studies suggest differential comparative safety across biologics in pediatric-onset disease, emphasizing the need to interpret pediatric signals separately from adult data [71].

JAK inhibitors require explicit thrombotic and cardiovascular risk framing, with a careful distinction between UC-specific trial/real-world evidence, extrapolation from rheumatoid arthritis (RA) regulatory safety signals, and dose-dependent risk. Regulatory warnings have been introduced after RA safety data (patients over 50 years old and at least one cardiovascular risk factor) demonstrated higher rates of pulmonary embolism and death with tofacitinib 10 mg twice daily compared with TNF antagonists, informing subsequent regulatory communications [73,74]. In contrast, UC-specific evidence indicates that VTEs are uncommon overall and, when observed, cluster during prolonged exposure and predominantly at 10 mg twice daily in patients with additional risk factors [55]. Most notably, in the tofacitinib UC clinical development program, VTEs were observed with 10 mg twice daily during long-term exposure, with no events reported for 5 mg twice daily [55,75]. Real-world cohort data in UC further suggest that comparative VTE risk versus TNF inhibitors may not be increased at either 5 mg or 10 mg twice daily, although point estimates may differ by dose and baseline risk profile [68]. Additionally, an IBD-focused systematic review and meta-analysis found no significant increase in MACE or VTE across JAK inhibitors in IBD trials overall, while reinforcing the importance of dose and patient risk stratification [76]. These considerations support careful patient selection, avoidance of prolonged high-dose maintenance therapy when alternatives exist, as well as de-escalation to 5 mg twice daily after induction when feasible, particularly in older patients with additional cardiovascular risk factors [76,77,78].

Overall, treatment-associated thrombotic risk in IBD appears driven predominantly by disease activity, systemic corticosteroid exposure, and baseline cardiovascular risk factors, rather than biologic class itself. Systemic corticosteroids demonstrate the most consistent treatment-associated VTE signal, including approximately threefold higher VTE occurrence in population data and markedly higher event rates compared with biologic-only strategies [22,58]. Anti-TNF therapy is associated with lower VTE rates compared with corticosteroids, likely reflecting both direct anti-inflammatory effects and successful disease control [54,64]. Vedolizumab and ustekinumab demonstrate generally favorable thrombotic and cardiovascular safety profiles in adult populations, though comparative estimates remain subject to confounding by indication and pediatric-onset CD signals for vedolizumab warrant further study [65,66,67,68,69,71,72]. For JAK inhibitors, the strongest safety signals derive from higher-risk RA populations treated with high-dose tofacitinib and are less consistent in UC-specific programs, where dose (10 mg twice daily) and patient risk profile appear to be the key modifiers of net thrombotic risk [55,73,74,76]. Taken together, these data reinforce that achieving and maintaining disease control with steroid-sparing strategies is a primary approach to reducing thrombotic risk in IBD, with drug-specific risk stratification most relevant for JAK inhibitors in higher-risk patients.

## 7. Clinical Management and Future Perspectives

Before initiating biologic or small-molecule therapy in IBD patients, an individualized risk assessment should be performed and integrated into clinical practice, especially considering the safety profiles of different therapeutic agents. Existing guidelines recommend screening for traditional risk factors, such as hypertension, diabetes, dyslipidemia, and smoking, particularly when considering advanced therapies [79]. The American Gastroenterological Association recommends cardiovascular risk stratification before initiating small-molecule therapy, especially JAK inhibitors, including baseline lipid profiles and regular monitoring [79]. Anti-TNF agents should be avoided in advanced congestive heart disease or demyelinating disease [79]. However, traditional risk factor calculators may underestimate the risk in young patients with high inflammatory activity.

Clinical management of thrombotic and cardiovascular risk in IBD should be embedded in routine care because risk is dynamic and it rises predictably with active inflammation and acute-care exposure. Among patients with IBD, hospitalization substantially increases the risk of VTEs, and VTE-associated mortality has been reported to be higher compared with non-IBD patients [80]. A practical summary of high-risk windows and recommended thromboprophylaxis is provided in Table 3.

Thus, current guidelines recommend pharmacologic thromboprophylaxis for all hospitalized patients with IBD, unless contraindicated. A prophylactic dose of low-molecular-weight heparin (LMWH) is preferred over unfractionated heparin in acutely and critically ill patients.

In ambulatory flares, routine prophylaxis is not advised, but ECCO supports considering temporary LMWH prophylaxis in patients with moderate-to-severe activity plus additional VTE risk factors until clinical improvement, noting that prophylactic LMWH has not been associated with increased major bleeding in typical practice [81].

Major IBD-related surgery represents a high-risk window for VTE and in-hospital prophylaxis should be ensured, with extended post-discharge prophylaxis being considered in selected high-risk patients (active disease, prolonged immobility, systemic corticosteroids, prior VTE) [81].

Recent guidelines recommend that outpatients with active IBD during the third trimester of pregnancy should receive pharmacological prophylaxis, unless contraindicated, and highlight the importance of prophylaxis following caesarean section [82].

According to existing guidelines, acute VTE in IBD patients should be managed using therapeutic-dose DOACs, with LMWH being an alternative when oral therapy is not suitable [81,82]. The duration of the anticoagulant therapy should be decided based on the type of VTE and the provoking factor. When a major transient risk factor is present, such as surgery, hospitalization, or a severe flare, a limited treatment course of 3 months should be performed, together with controlling the inflammatory activity of the IBD. For unprovoked events, extended anticoagulation should be discussed according to standard guidance, balancing recurrence risk against bleeding risk and patient preferences [81,82]. In practice, recurrence prevention depends not only on anticoagulant selection but also on achieving durable steroid-sparing disease control and minimizing repeated high-risk exposures—because inflammatory activity and systemic steroid burden repeatedly track with risk in IBD.

Cardiovascular prevention in IBD should generally follow established population frameworks—blood pressure, lipid and diabetes management, smoking cessation, physical activity, and weight optimization—while recognizing that systemic inflammation can act as a risk enhancer and may not be captured by conventional risk calculators [83]. Statins are considered safe and should be used according to general indications; there is no evidence to support IBD-specific antihypertensive regimens, and ACE inhibitors or angiotensin II receptor blockers remain appropriate first-line options. Low-dose aspirin for primary or secondary cardiovascular prevention does not appear to increase the risk of IBD exacerbations and can be used when indicated [83].

Even with guideline-based thromboprophylaxis during hospitalization and standard cardiovascular prevention, residual thrombotic and cardiovascular events continue to occur in IBD, largely because risk concentrates in predictable but dynamic windows—active inflammation, perioperative periods, and early post-discharge transitions. Accordingly, future efforts should complement context-based prophylaxis with mechanism-informed, individualized strategies that target key thromboinflammatory pathways linking intestinal disease activity to systemic vascular vulnerability.

Future risk stratification may be enhanced through integrated prediction models that integrate clinical exposures (hospitalization, surgery, corticosteroid exposure) with inflammatory biomarkers. Thus, dynamic monitoring of CRP and calprotectin may trace inflammation, with D-dimers reflecting the thrombotic risk during flares and treatment escalations or adjustments. Elevated D-dimer levels have been frequently associated with active IBD and predict VTE risk in hospitalized patients, although clear cut-off values remain undefined [84,85]. Artificial intelligence may support this approach by integrating longitudinal biomarkers, medication exposure (including corticosteroid use), admissions/surgery, and comorbidities into dynamic risk prediction and decision support tools [86].

When it comes to the pathophysiological links involved, experimental data revealed strategies to reduce TMAO and further attenuate platelet hyperreactivity through microbial choline TMA lyase inhibition [87,88]. Although these approaches have not yet been tested for vascular endpoints in IBD populations, they provide a biologically aligned framework for adjunctive risk reduction in selected phenotypes where platelet hyperreactivity and microbiota-dependent signaling appear promising. However, these strategies remain in early preclinical development and are not close to clinical translation.

Moreover, reducing systemic exposure to microbial products, targeting the LPS-TLR4 signaling, and further disrupting the TF induction, platelet activation, and endothelial dysfunction axis also provide valuable insights and potential therapeutic targets. Candidate agents that have been studied clinically in other inflammatory settings include the MD2–TLR4 antagonist eritoran and the small-molecule TLR4 signaling inhibitor resatorvid (TAK-242), both of which have extensive preclinical literature and human safety experience outside IBD [89]. However, neither of them has advanced to IBD-specific endpoint trials, and both are yet to be integrated into clinical practice.

Additionally, immunothrombosis-directed strategies are progressively studied, particularly due to targeting NET formation and degradation [90,91]. PAD4 inhibitors (e.g., GSK484, JBI-589) have shown proof-of-concept suppression of NET formation in inflammatory models, but no PAD4 inhibitor is currently ready for routine clinical use, underscoring the need for stepwise translation and rigorous safety assessment [91].

Beyond targeting specific immunothrombosis pathways, effective control of the underlying intestinal inflammation may provide one of the most sustainable strategies in reducing the thrombotic risk. Anti-TL1A monoclonal antibodies represent a promising new class of IBD therapeutics with the potential to achieve durable disease control and thereby interrupt the inflammation–thrombosis axis [92,93,94]. TL1A signaling through the DR3 (death-receptor 3) is upregulated in the intestinal tissue in IBD patients and amplifies the activity of Th1, Th17, further stimulating the production of pro-inflammatory cytokines (TNF-α, IL-1β, IL-6, IL-17, and IFN-γ) [92,93]. Phase 2 trials of anti-TL1A antibodies (including tulisokibart and afimkibart) have demonstrated significant improvements in clinical remission, endoscopic healing, and histologic outcomes in patients with moderate-to-severe ulcerative colitis and Crohn’s disease who failed conventional and advanced therapies [95,96]. Notably, anti-TL1A therapy reduces tissue inflammatory T cell and macrophage pathways, decreases peripheral inflammatory T cell cytokines, and modulates intestinal pathobionts, providing mechanistic evidence for broad immunomodulation [93,94]. Although no studies have directly assessed direct cardiovascular and thrombotic outcomes, a sustained inflammatory remission would be expected to reduce the chronic inflammatory burden sustaining the prothrombotic state, further mitigating the risk of VTEs in these patients. Among the mechanistic approaches discussed, anti-TL1A therapy is closest to clinical translation, with phase 3 trials ongoing and regulatory submissions anticipated [97].

## 8. Conclusions

IBD is associated with an increased risk of VTE and a modest risk of arterial cardiovascular complications, with event rates varying markedly by inflammatory activity and high-risk clinical contexts such as hospitalization, surgery, and perioperative care. Mechanistic data support an integrated thromboinflammatory framework in which gut dysbiosis and barrier dysfunction promote systemic innate immune activation, leading to coordinated tissue factor-dependent coagulation, platelet–leukocyte interactions, endothelial dysfunction, impaired fibrinolysis, and NET-mediated immunothrombosis that collectively lower the threshold for thrombosis.

Clinically, sustained, steroid-sparing control of inflammation remains central to cardiovascular risk reduction and should be complemented by guideline-based in-hospital thromboprophylaxis, individualized perioperative strategies, and selective post-discharge or outpatient prophylaxis in patients with high-risk profiles. Future progress will likely come from refined risk stratification across dynamic high-risk windows using clinical exposures and biomarker trajectories, and from adjunctive, mechanism-informed approaches that target key nodes at the gut–vascular interface, with the goal of reducing residual thrombotic and cardiovascular events beyond what can be achieved through conventional prevention alone.

## Figures and Tables

**Table 1:** Major studies assessing cardiovascular risk beyond VTE in IBD patients.

Author (Year)	Evidence Type (Number of Patients)	Cardiovascular Outcome	Key Findings
Thomas et al. (2025) [42]	Meta-analysis (2.2 million individuals)	Myocardial infarction	HR 1.29 (1.07–1.56); higher risk during flares and with active disease
		Ischemic heart disease	HR 1.16 (1.01–1.33); higher risk in women and with corticosteroid use
		Stroke	HR 1.15 (1.09–1.20); higher risk in CD, women, and during flares; excess risk persists ≥ 25 years
		MACE	HR 1.19 (1.09–1.30); risk increases with active disease
Sun et al. [43]	Population-based cohort (81.749 IBD patients)	Heart failure	aHR 1.19 (1.15–1.23); risk increases with histologic inflammation and corticosteroid use; excess risk persists ≥ 20 years
Sun et al. [44]	Population-based, sibling-controlled cohort (83.877 IBD patients)	Arrhythmias	aHR 1.15–1.30 across IBD subtypes; excess risk persists ≥ 25 years

HR: hazard ratio, CD: Crohn’s disease, aHR: adjusted hazard ratio, IBD: inflammatory bowel disease, MACE: major adverse cardiovascular events.

**Table 2:** Therapeutic agents in IBD and their thrombotic/cardiovascular effects.

Therapeutic Agent	Thrombotic or CV Risk	Quality of Evidence
Aminosalicylates (5-ASA)	VTE: no consistent increase CV: no consistent increase, potential long-term use associated with lower IHD risk in observational data, potentially confounded by disease severity/treatment selection	Moderate (mechanistic, observational, meta-analyses)
Corticosteroids	VTE: increased risk, dose- and context-dependent CV: potentially increased, correlated with prolonged and repeated use by indirect hypertension, dyslipidemia and insulin resistance	Moderate to high (large observational cohorts, meta-analyses)
Immunomodulators	VTE: overall neutral, no consistent increase reported CV: no consistent increase reported	Moderate (observational cohorts)
Anti-TNFα	VTE: lower than corticosteroids CV: no consistent increase reported Net effect likely favorable due to inflammatory control	Moderate to high (RCTs, large cohorts, meta-analyses)
Anti-integrin antagonists (Vedolizumab)	VTE: overall low signal, comparative estimates heterogeneous confounding by severity/steroids CV: no consistent increase reported Recommendation: VTE prophylaxis in high-risk periods	Moderate (trial safety, observational cohorts)
IL-12/23 Inhibitors (Ustekinumab)	VTE: overall neutral, no consistent increase reported CV: overall neutral, no consistent increase reported Better safety profile in pediatric populations	Moderate (trial safety, observational cohorts)
JAK inhibitors (Tofacitinib)	VTE and CV: regulatory warnings due to increased risk, dose- and patient-dependent UC-specific analyses show an absolute low Tofacitinib: approved for moderate-to-severe UC unresponsive to anti-TNFα therapy Recommendation: use the lowest effective dose; avoid prolonged high-dose maintenance when alternatives exist	Moderate (RCTs for UC, post-marketing and regulatory safety data, RA safety signal)

5-ASA, 5-aminosalicylates; CV, cardiovascular; IBD, inflammatory bowel disease; IHD, ischemic heart disease; JAK, Janus kinase; RCTs, randomized controlled trials; UC, ulcerative colitis; VTE, venous thromboembolism; RA, rheumatoid arthritis.

**Table 3:** Clinical settings, high-risk windows, and recommended thromboprophylaxis in IBD.

Clinical Setting	Risk Factors	Recommendation
Hospitalized IBD *	Active moderate–severe inflammation, immobility, dehydration, infection, systemic corticosteroids, prior VTE, cancer, obesity, older age	Pharmacologic thromboprophylaxis for all hospitalized IBD patients unless contraindicated; LMWH prophylaxis preferred over UFH in acutely/critically ill patients
Perioperative (major IBD-related surgery)	Major abdominal surgery, active inflammation, systemic corticosteroids, prolonged immobility, infection/SIRS, prior VTE, cancer	In-hospital LMWH prophylaxis + early mobilization; consider extended post-discharge prophylaxis in selected high-risk patients after major surgery
Ambulatory flare	Moderate–severe activity plus additional risk factors: prior VTE, immobility, dehydration, systemic corticosteroids, cancer	Not routinely recommended; consider temporary LMWH in very high-risk outpatients until clinical improvement
Pregnancy and postpartum	Active disease (especially late pregnancy), prior VTE/thrombophilia, obesity, immobility, systemic corticosteroids, caesarean delivery	Individualized LMWH prophylaxis in high-risk pregnancy settings; postpartum prophylaxis emphasized after caesarean section

* Refers to hospitalization due to flare or non-surgical disease. IBD, inflammatory bowel disease; VTE, venous thromboembolism; LMWH, low-molecular-weight heparin; UFH, unfractionated heparin; SIRS, systemic inflammatory response syndrome.

## Data Availability

No new data were created or analyzed in this study.

## Abbreviations

The following abbreviations are used in this manuscript:

5-ASA	5-aminosalicylates
ACE	angiotensin-converting enzyme
AF	atrial fibrillation
CD	Crohn’s disease
CI	confidence interval
DOACs	direct oral anticoagulants
DR3	death receptor 3
ECCO	European Crohn’s and Colitis Organisation
F VII	coagulation factor VII
F VIIa	activated coagulation factor VII
HR	hazard ratio
IBD	inflammatory bowel disease
IFN-γ	interferon gamma
IL-1β	interleukin 1 beta
IL-6	interleukin 6
IL-17	interleukin 17
IRR	incidence rate ratio
JAK	Janus kinase
LPS	lipopolysaccharide(s)
LMWH	low-molecular-weight heparin
MACE	major adverse cardiovascular events
MI	myocardial infarction
NETs	neutrophil extracellular traps
NO	nitric oxide
OCTAVE	Oral Clinical Trials for Tofacitinib in Ulcerative Colitis (trial program)
OR	odds ratio
PAD	peripheral artery disease
PAD4	peptidyl arginine deiminase 4
PAI-1	plasminogen activator inhibitor-1
PE	pulmonary embolism
RA	rheumatoid arthritis
ROS	reactive oxygen species
RR	relative risk
TF	tissue factor
Th17	T helper 17
TL1A	TNF-like ligand 1A
TLR4	Toll-like receptor 4
tPA	tissue plasminogen activator
TMAO	trimethylamine-N-oxide
TNF-α	tumor necrosis factor alpha
UC	ulcerative colitis
VTE	venous thromboembolism
α4β7	alpha-4 beta-7 integrin
